# Sodium-glucose cotransporter 2 inhibition prevents renal fibrosis in cyclosporine nephropathy

**DOI:** 10.1007/s00592-021-01681-2

**Published:** 2021-03-24

**Authors:** Giovanna Castoldi, Raffaella Carletti, Silvia Ippolito, Massimiliano Colzani, Francesca Barzaghi, Andrea Stella, Gianpaolo Zerbini, Gianluca Perseghin, Giovanni Zatti, Cira R. T. di Gioia

**Affiliations:** 1 Dipartimento Di Medicina E Chirugia, Università Degli Studi Di Milano-Bicocca, Via Cadore 48, 20900 Monza, Italy; 2 Dipartimento Di Scienze Radiologiche, Oncologiche E Anatomopatologiche, Istituto Di Anatomia Patologica Sapienza Universita’ Di Roma, Roma, Italy; 3 Laboratorio Analisi Chimico Cliniche, Ospedale San Gerardo, ASST Monza, Monza, Italy; 4 Unita’ Complicanze del Diabete, Diabetes Research Institute, IRCCS Istituto Scientifico San Raffaele, Milano, Italy; 5 Dipartimento Di Medicina Interna E Riabilitazione, Policlinico Di Monza, Monza, Italy; 6 Clinica Ortopedica, Ospedale San Gerardo, ASST Monza, Monza, Italy

**Keywords:** Cyclosporine nephropathy, SGLT-2 inhibitors, Experimental models, Renal fibrosis, Rats

## Abstract

**Aims:**

Sodium-glucose cotransporter 2 (SGLT2) inhibitors, a new class of antidiabetic drugs, are nephroprotective in case of diabetes, but whether a similar beneficial effect may be detectable also in case of chronic non-diabetic kidney diseases remains still unknown. The aim of this study was to evaluate the effects of empagliflozin, a SGLT-2 inhibitor, on the progression of cyclosporine nephropathy, in the absence of diabetes.

**Methods:**

Sprague Dawley rats (*n* = 27) have been fed with low-salt diet starting 10 days before the beginning and finished at the end of the experimental period. Cyclosporine-A (CsA, 15 mg/kg/day, intraperitoneal injection, *n* = 8) and CsA plus empagliflozin (Empa, 10 mg/kg/day, per os, *n* = 7) were administered for 4 weeks. The control groups were treated with placebo (Control, *n* = 7) or empagliflozin (Control + Empa, *n* = 5). Blood pressure (plethysmographic method) was measured at the beginning and at the end of the experimental period. At the end of the experimental protocol, the kidneys were excised for histomorphometric analysis of renal fibrosis and for immunohistochemical evaluation of inflammatory infiltrates (monocytes/macrophages), type I and type IV collagen expression, and tyrosine hydroxylase expression, used as marker of sympathetic nerve activity.

**Results:**

CsA-treated rats showed a significant increase (*p* < 0.01) in blood pressure, which was reduced by administration of empagliflozin (*p* < 0.05). CsA administration caused an increase in glomerular and tubulo-interstitial fibrosis (*p* < 0.05), renal inflammatory infiltrates (*p* < 0.05), type I and type IV collagen expression (*p* < 0.01), and tyrosine hydroxylase expression (*p* < 0.01) as compared to the control rats and control + Empa-treated rats. Treatment with empagliflozin in CsA-treated rats reduced glomerular (*p* < 0.01) and tubulo-interstitial fibrosis (*p* < 0.05), type I and type IV collagen expression (*p* < 0.01), inflammatory cell infiltration (*p* < 0.01) and tyrosine hydroxylase expression (*p* < 0.05), as compared to rats treated with CsA.

**Conclusion:**

Empagliflozin administration caused a reduction in blood pressure in CsA-treated rats and showed a protective effect on CsA nephropathy by decreasing renal fibrosis, type I and type IV collagen expression, macrophage infiltration and tyrosine hydroxylase expression. These data suggest that empagliflozin promotes nephroprotection also in non-diabetic kidney disease.

## Introduction

Cyclosporine A (CsA) is a calcineurin inhibitor widely used from many years in organ transplantation [1, 2] and in the treatment of several autoimmune disorders [2, 3]. Although CsA treatment has improved the survival in transplanted patients, its use is limited by its potential nephrotoxicity and by the development of post-transplant hypertension [4, 5], which is a common clinical problem in transplanted patients.

It is well known for a long time that in renal transplant recipients chronic CsA nephropathy is a major cause of chronic allograft dysfunction [6], and it is characterized by tubular atrophy, inflammatory cell infiltration and hyalinosis of the afferent arterioles, followed by the development of renal fibrosis, mostly tubulo-interstitial fibrosis, which represents a strong predictor of end-stage renal disease [7]. Independently on the etiology, renal fibrosis, characterized by the increase in collagen and extracellular matrix protein deposition in glomerular and tubulo-interstitial area, represents a severe complication in case of chronic kidney diseases, leading to end-stage renal failure [8, 9].

Clinical trials have shown that sodium–glucose cotransporter 2 (SGLT2) inhibitors reduce the progression of kidney disease in type 2 diabetic patients at high cardiovascular risk with preserved renal function [10–12] or with different degrees of renal impairment, regardless of baseline renal function [13, 14].

The beneficial effects of SGLT2 inhibitors demonstrated in diabetic patients cannot be explained only by their glucose-lowering and metabolic effects, but it is likely associated with the pleiotropic effects of these drugs on the cardiovascular and renal system, which involved hemodynamic, cellular and molecular mechanisms [15–20].

Whether SGLT2 inhibitors could also be effective, and in particular protective, in case of non-diabetic kidney diseases remains unknown [21].

In fact, in non-diabetic preclinical models of nephropathies, dapagliflozin, a SGLT2 inhibitor, reduced proteinuria and podocyte damage [22], and empagliflozin, a SGLT2 inhibitor, improved renal function in obese Zucker rats [23], reduced renal dysfunction and fibrosis in unilateral ureteric obstruction [24], regulated the circadian rhythm of blood pressure in salt-treated obese rats [25], and finally prevented the development of renal fibrosis through an anti-inflammatory mechanism in Ang II-dependent hypertension [26]. Conversely, empagliflozin did not show nephroprotection in a murine model of oxalate-related nephrocalcinosis [27] and dapagliflozin did not modify proteinuria and renal fibrosis in 5/6 subtotally nephrectomized rats [28].

To clarify these issues, in the present study we investigated the role of SGLT2 inhibition in the progression of CsA nephropathy.

## Methods

### Experimental protocol

Animal husbandry was in conformity with the Institutional Guidelines in compliance with National laws and policies (D. L. n. 116, Gazzetta Ufficiale della Repubblica Italiana, suppl. 40, Feb. 18, 1992), and experiments were performed in accordance with the Guide for the Care and Use of Laboratory Animals published by the US National Institutes of Health (NIH Publication No. 85–23, revised 1996).

**Fig. 1:**
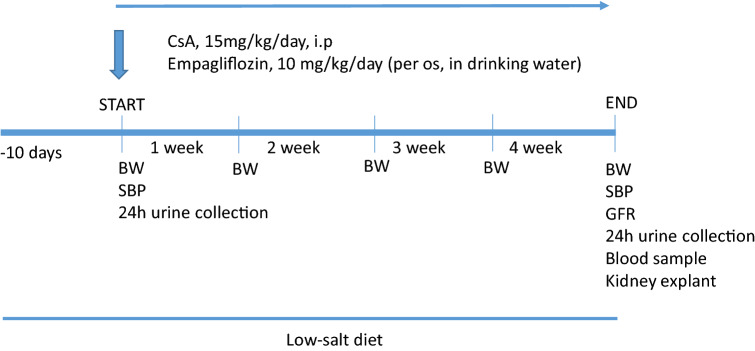
Scheme of the experimental protocol. Daily, CsA was administered by i.p. injection, and empagliflozin was provided per os in drinking water. CsA and empagliflozin administration were simultaneously started. Body weight (BW) was measured weekly. At the beginning and at the end of the experimental period, 24-h urine samples were collected and systolic blood pressure (SBP) was measured by the tail cuff method. At the end of the protocol, glomerular filtration rate (GFR) was measured and a blood sample was taken. Rats were euthanized by an overdose of anesthesia, and the kidneys were immediately excised

Conscious male Sprague Dawley rats, 150–200 g. body weight, 8–9 weeks old (Charles River, Calco, Italy), were individually housed in cages (or metabolic cages as necessary to collect 24-h urine samples) in a temperature-controlled room (22 °C) with a 12:12 light–dark cycle and free access to low-salt diet (Teklad 7034) and tap water [29]. Low-salt diet was used to increase renal cyclosporine damage [30, 31], starting 10 days before and during the entire experimental period (Fig. 1).

Body weight (BW, g) was measured weekly, and 24-h urine samples were collected at the beginning and at the end of the experimental protocol (Fig. 1). Systolic blood pressure (SBP, mmHg) was measured at the beginning and at the end of the experimental period by the tail cuff method (average of 6 recordings. BP Recorder, Ugo Basile Instruments, Italy) by an investigator who was unaware of the specific treatments [32] (Fig. 1).

Sprague Dawley rats (*n* = 27) were divided into four experimental groups. CsA (Sandimmune, Novartis) was administered at the dose of 15 mg/kg/day by intraperitoneal injection for 4 weeks to induce nephropathy (CSA-treated rats, *n* = 8). Empagliflozin (Empa, 10 mg/kg/day, dissolved in drinking water) was administered to CSA-treated rats (CSA + Empa-treated rats, *n* = 7). Control rats received placebo (*n* = 7) and another group was treated with empagliflozin (Control+Empa-treated rats, *n* = 5).

CsA administration and empagliflozin treatment started simultaneously (Fig. 1).

At the end of the protocol, glomerular filtration rate (ml/min/100 mg.kidney weight) was evaluated as creatinine clearance. Non-fasting plasma glucose (mg/dL), creatinine (mg/dL), sodium (mEq/L), calcium (mg/dL) and phosphate (mg/dL), and 24-h urinary glucose (mg/24 h), sodium (mEq/24 h), creatinine (mg/L), calcium (mg/dL) and phosphate (mg/dL) were measured by colorimetric technique on Cobas Roche (Mannheim, Germany). Plasma Rat Tartrate-Resistant Acid Phosphatase 5b (TRACP-5b, ng/ml) was measured by the Elisa method (Fine Test FN. Wuhan. China), following manufacturer’s instruction.

At the end of the experimental period, rats were euthanized by an overdose of anesthesia. The kidneys were immediately excised, weighted and sectioned longitudinally. The kidneys were fixed with 10% formalin, embedded in paraffin and used for light microscopic examination, immunohistochemistry and morphometric analysis to evaluate glomerular, tubulo-interstitial and perivascular fibrosis [29].

### Histological analysis and morphometric evaluation of renal fibrosis

Hematoxylin–eosin (H&E)-stained transmural renal sections (3 µm) were used to evaluate morphological changes with a Leica light microscope (Leitz Camera, Wetzlar, Germany) using a semiquantitative score based on severity and extension (negative, mild and focal, moderate and multifocal, severe and diffuse) by two experienced pathologists (C.R.T. di G. and R.C.). The collagen-specific Sirius Red staining was used for morphometric analysis of renal fibrosis. All slides with Sirius Red-stained renal sections were captured with Aperio scanner (Leica Biosystems), and then, twenty randomly selected images were analyzed to evaluate collagen volume fraction in glomerular, tubulo-interstitial and perivascular areas with a computerized imaging software (Image J, NIH, Bethesda, MD), as previously described [29]. Two independent investigators, blinded to the experimental protocol, performed image analysis.

The glomerular and tubulo-interstitial collagen volume fractions were evaluated at 20X magnification, respectively, in semi-automated fashion in 20 traced glomeruli (considered as matrix, cells, capillary loops and space surrounding glomerular segments) and in automated fashion in 20 fields without vessels or glomeruli, randomly selected from each kidney section. The glomerular collagen fraction was expressed as the ratio of red-stained collagen area to glomerular area, while the tubulo-interstitial collagen fraction as the ratio of collagen area to total area [32].

The perivascular collagen volume fraction was evaluated at 20X magnification in semi-automated fashion in 20 randomly selected intraparenchymal vessels from each kidney section. Only the collagen immediately surrounding each intraparenchymal vessel was considered to represent perivascular collagen deposition. The perivascular collagen fraction was expressed as the ratio between collagen area surrounding the traced vessel and total cross-sectional area, in order to correct differences in vessel size [29].

### Immunohistochemical evaluation of renal type I and type IV collagen expression

Immunohistochemical evaluation was performed on consecutive sections (3 μm) of formalin-fixed, paraffin-embedded renal tissue. Sections were deparaffinized in xylene and rehydrated through graded alcohol series. Endogenous peroxidase activity was blocked by 3% hydrogen peroxide. The sections were treated with microwave (pH 6 citrate buffer). The sections were then incubated at 4 °C overnight with type I collagen (1:300, rabbit anti-rat polyclonal antibody, Millipore Chemicon, Temecula, CA, USA, AB755P) or type IV collagen (1:150, rabbit anti-human polyclonal antibody, Abcam, Cambridge, UK, AB6586). The reaction was amplified with LSAB2+System-HRP (Dako CA, USA). A positive immunoreaction was identified after incubation with 3,3′-diaminobenzidine (DAB) and counterstaining with Mayer hematoxylin. Negative controls were obtained omitting the primary antibody. Sections were viewed using a Leica microscope (Leitz Camera, Wetzlar, Germany). Two independent investigators, blinded to the treatment, analyzed the immunostaining with a Leica microscope (Leitz Camera, Wetzlar, Germany), and subsequently, all immunostaining slides were captured with Aperio scanner (Leica Biosystems). The collagen immunostaining on randomly selected images was quantified using a computerized imaging software (Image J, NIH, Bethesda, MD), respectively, in semi-automated fashion in 20 traced glomeruli (40X magnification) and in automated fashion in 20 tubulo-interstitial fields (without vessels or glomeruli) (20X magnification), randomly selected from each kidney section. Type I and type IV collagen immunostaining was expressed as percentage (ratio of immunostaining area to glomerular or total area) [26, 29, 32].

### Immunohistochemical evaluation of renal monocyte/macrophage infiltration

The count of monocytes/macrophages infiltration was performed on formalin-fixed and paraffin-embedded transmural renal sections (3 μm), using a monoclonal mouse anti-rat monocytes/macrophage (CD68, clone ED1, MAB 1435, Chemicon, Temecula, CA). The histological sections were deparaffinized and rehydrated, treated by boiling in citrate buffer (0.01 mol/l, pH 6) in microwave (750 W), and incubated over night at 4 °C with primary antibody (1:300). The reaction product was amplified by Ultra Tek HRP Staining System (Scy TeK Laboratories, Utah, USA) and visualized with 3,3′-diaminobenzidine (DAB) (Dako, Glostrup, Denmark). Negative control was obtained by omitting the primary antibody. Sections were viewed using a Leica microscope (Leitz Camera), and all slides with immunostained renal section for each sample were captured with Aperio scanner (Leica Biosystems). Ten randomly selected images/section at X20 magnification were analyzed. Two independent pathologists blinded to the treatment counted the CD68-positive cells and took the average. The macrophages were expressed as mean value of positive cells/fields [29, 32].

### Immunohistochemical evaluation of renal tyrosine hydroxylase expression

The tyrosine hydroxylase expression was evaluated on formalin-fixed and paraffin-embedded renal sections (3 µm) using an anti-rabbit Anti-Tyrosine Hydroxylase antibody—Neuronal Marker (ab112 Abcam, Cambridge, UK). The sections were deparaffinized and rehydrated, treated with Proteinase K (20 µg/ml; Qiagen, Hilden, Germany) for 10 min at 37 °C and successively incubated with primary antibody (1:1000, for an hour). The reaction product was amplified by Ultra Tek HRP Staining System (Scy TeK Laboratories, Utah, USA) and visualized with 3,3′-diaminobenzidine (DAB) (Dako, Glostrup, Denmark). Negative control was obtained by omitting the primary antibody. Sections were viewed using a Leica microscope (Leitz Camera), and all slides with immunostained renal section for each sample were captured with Aperio scanner (Leica Biosystems). Ten randomly selected images/section at X20 magnification were analyzed by two independent investigators blinded to the treatment. The tyrosine hydroxylase immunostaining at nerve fibers was expressed as % (immunostaining area/total histological area) [33].

### Statistical analysis

Data are presented as means ± SEM. Differences among the groups of rats (Control, Control + Empa, CsA and CsA + Empa-treated rats) for systolic blood pressure, plasma glucose, calcium and phosphate, tartrate-resistant acid phosphatase-5b, body weight, kidney weight, kidney/body weight, glomerular filtration rate, 24-h diuresis and 24-h urinary sodium, glucose, calcium and phosphate excretion, renal glomerular, tubulo-interstitial and perivascular fibrosis, type I and type IV collagen expression, renal inflammatory infiltrates and tyrosine hydroxylase expression were assessed with the use of analysis of variance (ANOVA) followed by the Fisher’s protected least-significant test for post hoc comparisons. Differences between means were considered significant at *p* < 0.05.

## Results

### Effects of empagliflozin administration on systolic blood pressure, serological and urinary excretory parameters in CsA-treated rats

At the end of the experimental period, the effects of empagliflozin administration on systolic blood pressure, non-fasting plasma glucose, sodium, calcium, phosphate, tartrate-resistant acid phosphatase-5b, glomerular filtration rate, body weight, kidney weight, kidney/body weight ratio in CsA-treated rats are shown in Table 1. SBP was significantly higher in the CsA-treated rats and CsA + Empa-treated rats as compared with the Control and Control + Empa-treated animals. In CsA-treated rats, empagliflozin administration caused a significant decrease in blood pressure as compared with CsA-treated rats (Table 1).

**Table 1:** Systolic blood pressure (SBP, mmHg), non-fasting plasma glucose (mg/dL), sodium (mEq/L), calcium (mg/dL), phosphate (mg/dL), tartrate-resistant acid phosphatase-5b (TRACP-5b, ng/ml), glomerular filtration rate (GFR, ml/min/100 mg. kidney weight), body weight (BW, g.), kidney weight (g.), kidney/body weight ratio (mg/g), in Control, Control + Empa, CSA-treated rats and CSA + Empagliflozin-treated rats at the end of the experimental period

	Control	Control + Empa	CsA	CsA + Empa
SBP, mmHg	139.9 ± 5.3	145.9 ± 1.6	166.6 ± 3.3†*ε*	152.5 ± 3.1*δ
Plasma glucose, mg/dL	133.2 ± 9.0	142.6 ± 3.5	149.5 ± 11.3	115.7 ± 6.9 *δ*
Plasma sodium, mEq/L	141.8 ± 1.0	141.0 ± 0.8	143.2 ± 0.3	142.6 ± 0.8
Plasma calcium, mg/dL	5.42 ± 0.14	6.13 ± 0.49	7.16 ± 0.38†	5.77 ± 0.34 *δ*
Plasma phosphate, mg/dL	6.49 ± 0.12	6.23 ± 0.17 *δ*	7.00 ± 0.33	5.70 ± 0.23 *ζ*
Plasma TRACP-5b, ng/ml	0.669 ± 0.04	0.701 ± 0.01	0.854 ± 0.04*	0.827 ± 0.06*
GFR, ml/min/100 mg.Kidney	0.290 ± 0.02	0.243 ± 0.01	0.220 ± 0.01*	0.253 ± 0.02
BW, g	405.8 ± 14.2	374.2 ± 6.2	398.3±9.6	348.5 ± 14.3† *ζ*
Kidney weight, g	1.475 ± 0.03	1.635 ± 1.33	1.571 ± 0.07	1.583 ± 0.06
Kidney/Body weight, mg/g	3.647 ± 0.10	4.386 ± 0.41†	3.938 ± 0.12	4.551 ± 0.10† *δ*

* = *p* < 0.05 versus control; † = *p* < 0.01 versus control; δ = *p* < 0.05 versus CSA; ζ = *p* < 0.01 versus CSA; *ε* = *p* < 0.01 versus Control + Empa

Even though plasma glucose level remains within normal range for non-fasting conditions, its concentration was increased in CsA-treated rats as compared with control rats. Empagliflozin administration did not significantly modify plasma glucose in control rats, but it was able to control the increase in plasma glucose in CsA-treated rats (Table 1). In CsA-treated rats, plasma sodium and phosphate did not change significantly, while plasma calcium and tartrate-resistant acid phosphatase-5b, marker of osteoclast activity, were significantly increased as compared to control rats. Empagliflozin administration did not modify plasma sodium, calcium, phosphate and tartrate-resistant acid phosphatase-5b in Control + Empa-treated rats, but it prevented the increase in plasma calcium in CsA-treated rats and reduced plasma phosphate as compared with CsA-treated rats, while it did not modify the increase of tartrate-resistant acid phosphatase-5b caused by CsA administration (Table 1).

Empagliflozin administration did not modify glomerular filtration rate in control rats. Glomerular filtration rate was significantly decreased in CsA-treated rats, but not in CsA + Empa-treated rats, as compared with control rats.

Body weight was similar in CsA-treated and control, while in CsA + Empa-treated rats body weight was lower as compared with control and CsA-treated rats (Table 1). No differences in kidney weight were observed in the different experimental groups. CsA administration did not modify kidney weight/body weight ratio as respect to control rats. Empagliflozin administration caused a significant increase in kidney weight/body weight ratio in control + Empa-treated rats as compared with control rats. CsA + Empa-treated rats showed a significant increase in kidney weight/body weight ratio as compared with both control and CsA-treated rats (Table 1).

**Fig. 2:**
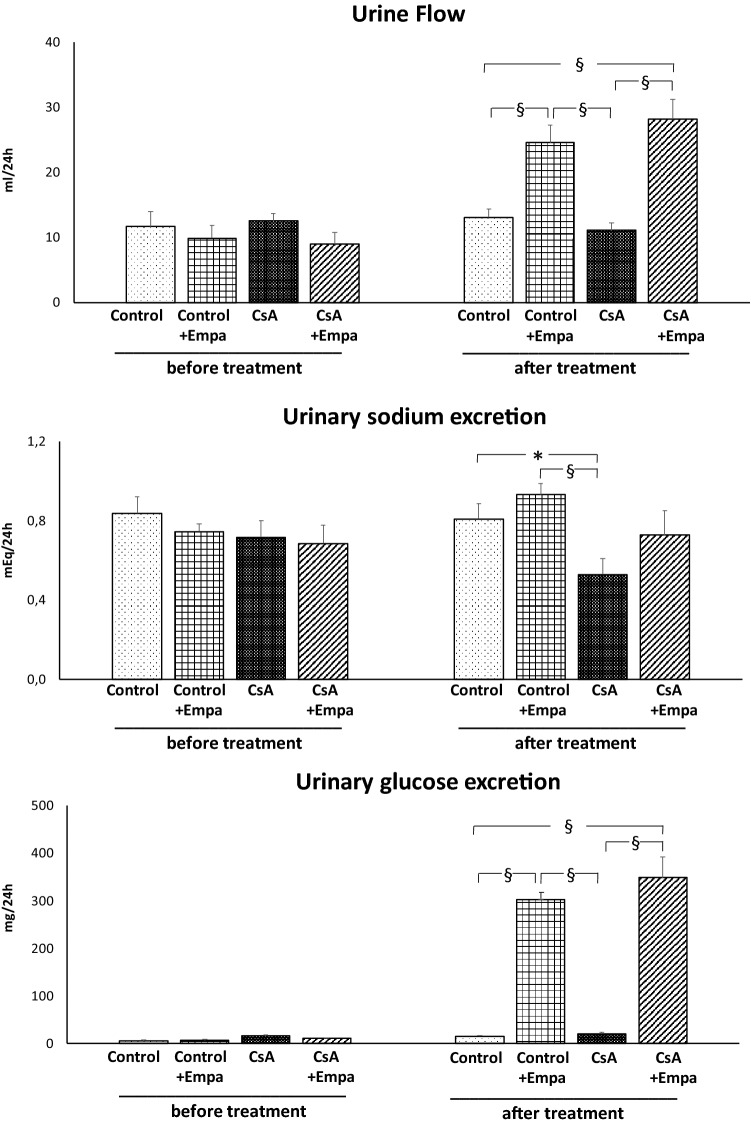
Effects of CsA and empagliflozin administration on 24-h urine flow, urinary sodium excretion and urinary glucose excretion before and at the end of the experimental protocol treatments. *: *p* < 0.05; §: *p* < 0.01

**Fig. 3:**
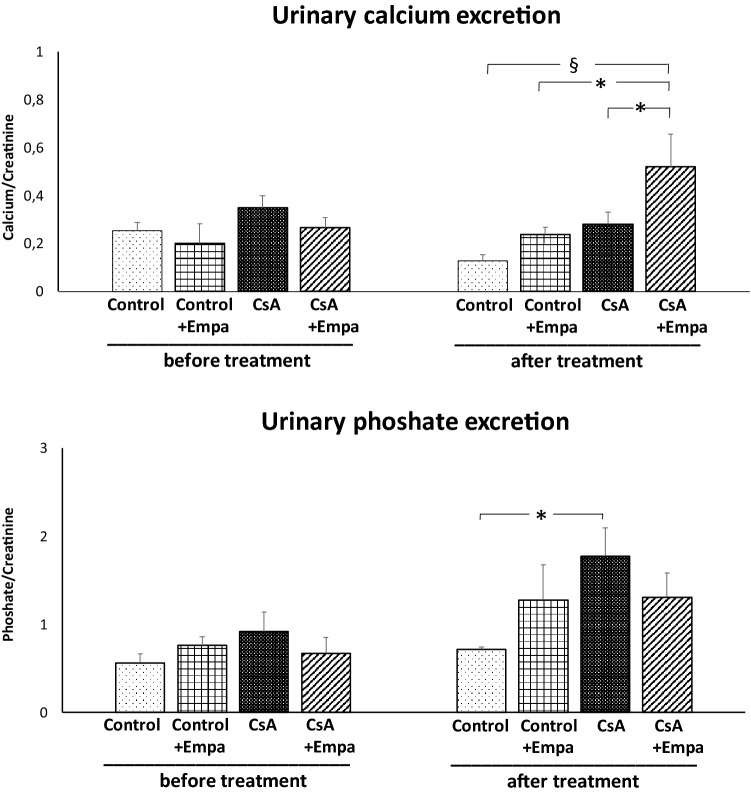
Effects of CsA and empagliflozin administration on urinary calcium and phosphate excretion during the 24 h before and at the end of the experimental protocol treatments. *: *p* < 0.05; §: *p* < 0.01

Before starting the treatments, urine flow and urinary sodium, calcium and phosphate excretion were similar among the experimental groups and urinary glucose excretion was absent (Figs. 2 and 3). CsA treatment did not modify urine flow and glucose excretion, but decreased sodium excretion as compared to control rats (Fig. 2). Empagliflozin administration in control rats and in CsA-treated rats caused an increase in diuresis and glycosuria, and, as a result, in CsA-treated rats urinary sodium excretion became closer to the one observed in the control rats (Fig. 2). CsA administration resulted in a slight, even though not significant increase in the urinary calcium excretion, but it caused a significant increase in urinary phosphate excretion (Fig. 3). Empagliflozin administration caused an increase in urinary calcium excretion in CsA-treated rats as compared to CsA, control and Control + Empa-treated rats. CsA administration increases urinary phosphate excretion, which was blunted by empagliflozin treatment (Fig. 3).

### Effects of empagliflozin treatment on renal fibrosis, type I and type IV collagen expression, inflammatory cell infiltration and tyrosine hydroxylase expression in CsA-treated rats

**Fig. 4:**
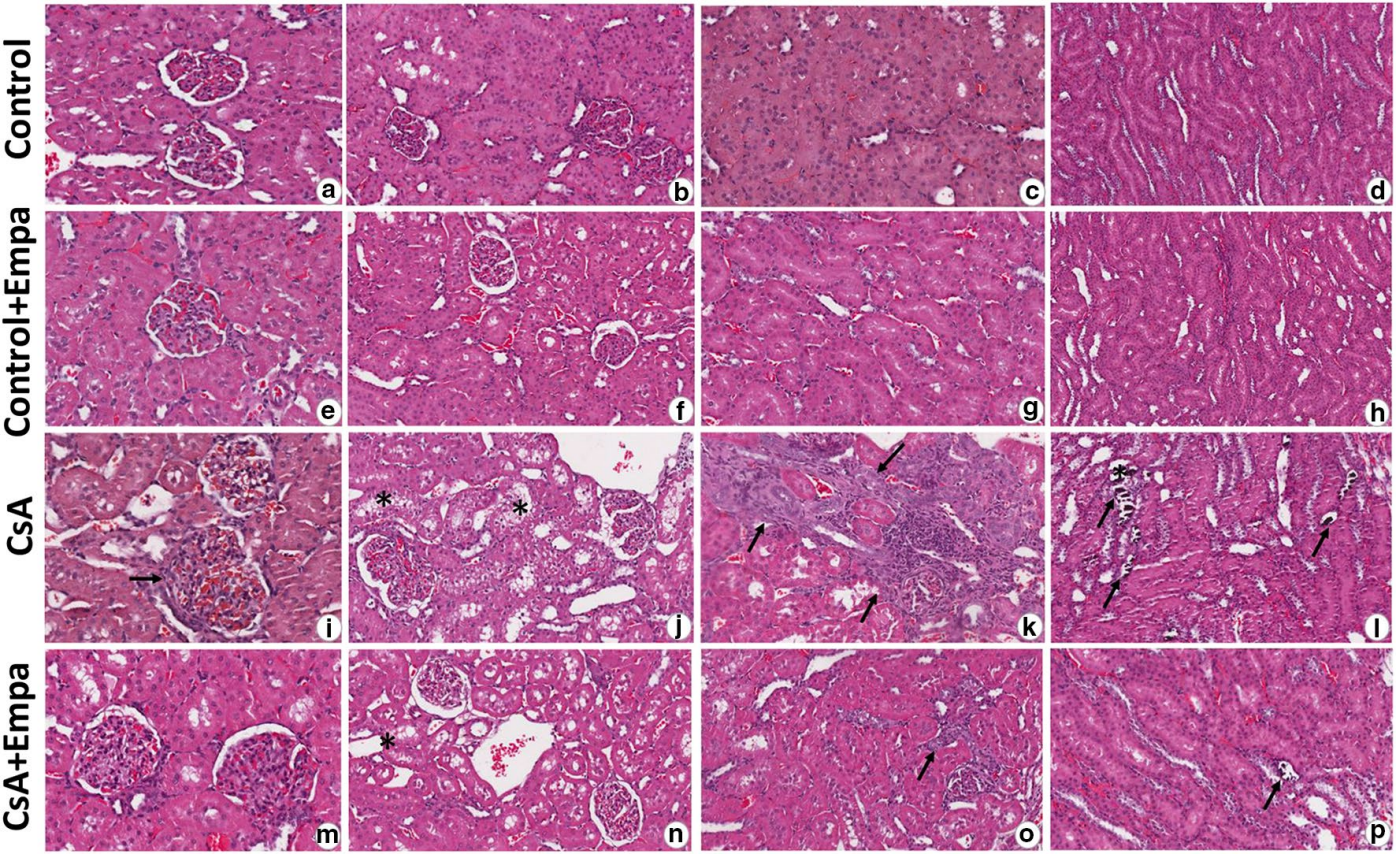
Representative photomicrographs of renal tissue histological changes in CsA (i–l) and CsA + empagliflozin-treated rats (m–p) versus Control (a–d) and Control + Empa (e–h) groups. In CsA-treated rat group, H&E-stained renal sections show wider glomeruli with dilated capillaries full of red blood cells and dilated Bowman’s capsules (i, arrow), tubular isometric vacuolization of proximal tubular segments (j, asterisk), tubulo-interstitial fibrosis associated with tubular atrophy and interstitial inflammatory infiltration (k, arrows), and dystrophic microcalcifications (l, arrows). These renal changes were expressed only focally in CsA + Empaglifozin-treated rat group (m–p, asterisk and arrows). Both control (a–d) and control + empaglifozin (e–h) groups showed normal histological architecture concerning glomeruli and tubules. Hematoxylin–eosin (H&E). Original magnification X20: b, c, d, f, g, h, j, k, l, n, o, p; X40: a, e, i, m.

The kidneys of the CsA-treated rat group showed significant histopathological changes related to calcineurin inhibitor toxicity. In fact, in this group the H&E-stained renal sections showed multifocal moderate changes as tubular injury with isometric vacuolization of proximal tubular segments, tubular atrophy, some dystrophic microcalcifications, interstitial inflammatory infiltration and tubulo-interstitial fibrosis. The glomeruli were wider with dilated capillaries full of red blood cells and dilated Bowman’s capsules, focally fibrotic (Fig. 4). These renal changes were mild and expressed focally in CsA + empagliflozin-treated rat group (Fig. 4). Both control and CsA + empagliflozin groups showed normal histological architecture concerning glomeruli and tubules (Fig. 4).

**Fig. 5:**
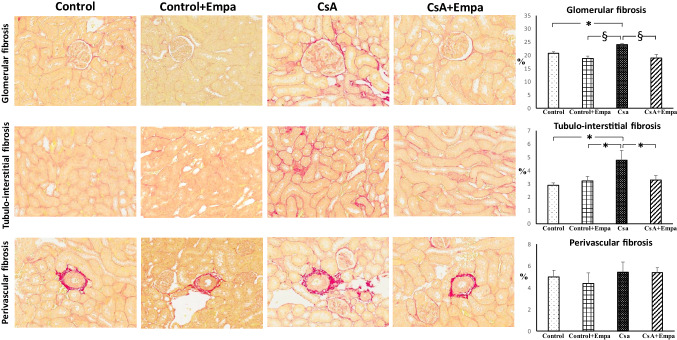
Effect of empagliflozin administration on glomerular, tubulo-interstitial and perivascular fibrosis in CsA-treated rats.*On the left*. Representative photomicrographs of glomerular, tubulo-interstitial and perivascular fibrosis in control, control + Empa, CsA, and CsA + empagliflozin-treated rats (original magnification X20). Sirius red, collagen-specific staining, shows an increase in glomerular and tubulo-interstitial fibrosis in CsA-treated rats, as compared to control and control + Empa-treated rats. Empagliflozin administration prevented the increase in glomerular and tubulo-interstitial fibrosis in CsA-treated rats. Perivascular fibrosis did not change among the different experimental groups.*On the right.* Quantification of glomerular, tubulo-interstitial and perivascular fibrosis in the different groups of rats. Data are means ± SEM. *: *p* < 0.05. §: *p* < 0.01

The Sirius Red-stained sections showed a significantly increased glomerular and tubulo-interstitial fibrosis in CsA-treated rats, while perivascular fibrosis was not modified (Fig. 5). Empagliflozin administration prevented the increase in glomerular and tubulo-interstitial fibrosis in CsA-treated rats (Fig. 5).

**Fig. 6:**
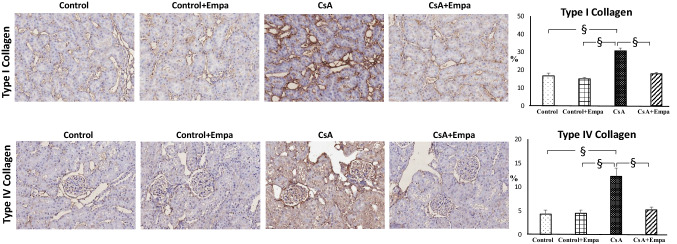
Effect of empagliflozin administration on type I and type IV collagen expression in CsA-treated rats. *On the left*. Representative photomicrographs of type I and type IV collagen expression in control, control + Empa, CsA, and CsA + empagliflozin-treated rats (original magnification X20). Type I collagen showed an increased tubulo-interstitial staining in CsA-treated rats, while type IV collagen was increased mainly in the intraglomerular area in CsA-treated rats, as compared to control and control + Empa-treated rats. Empagliflozin administration prevented the increase of both type I and type IV collagen in CsA-treated rats. *On the right*. Quantification of type I and type IV collagen proteins in renal tubulo-interstitial and glomerular area, respectively, in the different groups of rats. Data are means ± SEM. §: *p* < 0.01.

CsA administration increased both type I and type IV collagen expression, located mainly at tubulo-interstitial and glomerular level, respectively (Fig. 6). Empagliflozin administration prevented the increase of type I and type IV collagen expression in CsA-treated rats (Fig. 6).

**Fig. 7:**
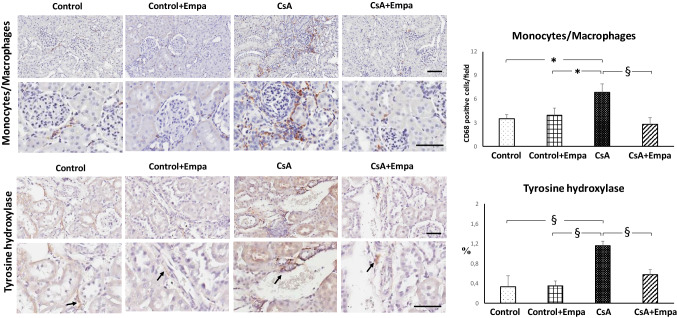
Effects of empagliflozin administration on renal inflammatory cell infiltration and tyrosine hydroxylase expression in CsA-treated rats. *On the left.* Immunohistochemical identification of interstitial inflammatory cell infiltration and tyrosine hydroxylase expression in kidney of control, control + Empa, CsA and CsA + Empa-treated rats. The immunostaining showed a significant increase in interstitial monocyte/macrophage cells (brown reaction) and tyrosine hydroxylase expression at intraparenchymal nerve fiber level (brown reaction) in CsA-treated rats as compared with control and control + Empa-treated rats. Empagliflozin administration prevented the increase of monocyte/macrophage infiltration and tyrosine hydroxylase expression in CsA-treated rats. Immunoperoxidase stain, in both the panels original magnification upper X20 and inferior X40. *On the right*: Quantification of staining of renal monocyte/macrophage inflammatory cells and tyrosine hydroxylase expression in the different groups of rats. Data are means ± SEM. *: *p* < 0.05. §: *p* < 0.01

A significant increase in monocyte/macrophage infiltration, localized in the tubulo-interstitial area and tyrosine hydroxylase expression at the level of the intraparenchymal sympathetic nerve fibers, has been observed in CsA-treated rats when compared to control rats (Fig. 7). Empagliflozin administration completely blocked inflammatory cell infiltration in the renal parenchyma and renal tyrosine hydroxylase expression caused by CsA administration (Fig. 7).

## Discussion

The results of this study demonstrate that chronic empagliflozin treatment promotes nephroprotection in CsA nephropathy, by reducing the increase in blood pressure caused by CsA administration and by preventing renal fibrosis, inflammatory cell infiltration and tyrosine hydroxylase expression, used as marker of sympathetic nerve activity.

In our experimental conditions, CsA administration caused an increase in blood pressure and renal fibrosis at glomerular and tubulo-interstitial area, but not at perivascular level, and an increase in renal inflammatory cell infiltration and tyrosine hydroxylase expression. Empagliflozin treatment blunted the increase in blood pressure caused by CsA administration as compared to CsA-treated rats, but in CsA + Empa-treated rats blood pressure remained higher than in control rats. In CsA-treated rats, empagliflozin administration prevented the development of glomerular and tubulo-interstitial fibrosis and blunted renal inflammatory cell infiltration and tyrosine hydroxylase expression.

Hypertension and nephrotoxicity represent the main complications during the treatment with calcineurin inhibitor [4–6].

Different mechanisms contribute to the pathogenesis of hypertension caused by CsA treatment, as vasoconstriction [34], sodium retention [35] and the activation of the sympathetic nervous system [36]. CsA-induced sodium retention is caused by the activation of the sympathetic nervous system [37], and the activation of catecholamine synthetic pathway is involved in blood pressure increase caused by CsA [36]. CsA treatment may cause an acute reduction of glomerular filtration rate and renal blood flow, which might be reversible in the case of acute nephrotoxicity, or irreversible in the chronic CsA nephropathy. All these mechanisms may be modulated by SGLT2-inhibition and are suggested to explain the nephroprotective action of SGLT2 inhibitors in diabetes, beyond the beneficial effect due to the reduction of hyperglycemia [15–20].

In fact, an increase in urine flow and urinary sodium excretion [38] has been suggested to explain the reduction of blood pressure observed in the clinical trials in type 2 diabetic patients [39–41], with positive effects on nephroprotection. In the experimental model of CsA nephropathy, which is characterized by a moderate increase of blood pressure, sustained by the activation of sympathetic activity and by sodium retention, we have observed a reduction of blood pressure values in CsA + empagliflozin-treated rats as compared with CsA-treated rats, in the presence of an increase of diuresis and glycosuria. In addition, while in CsA-treated rats urinary sodium excretion was lower as compared to control rats, indicating sodium retention caused by CsA treatment, empagliflozin administration in CsA-treated rats reverted urinary sodium excretion toward control values (Fig. 1). These results might explain the pathophysiological mechanisms underlying the reduction of blood pressure in CsA-treated rats during the treatment with SGLT2 inhibitors. Interestingly, while in Ang II-dependent hypertension we did not detect a significant reduction of blood pressure following empagliflozin administration, despite a significant increase of diuresis and natriuresis [26], in the experimental model of CSA nephropathy we observe that empagliflozin administration causes a significant decrease in blood pressure, which, however, remained higher than the control group values. We can attribute this difference to the fact that in the CSA nephropathy model the increase in blood pressure reaches moderate values, which may be affected by the diuretic effect induced by the SGLT2 inhibition. Conversely, the experimental model of Ang II-dependent hypertension is reasonable more ‘aggressive’ than the one induced by CsA and therefore less suitable to be affected by the diuretic and natriuretic action caused by empagliflozin treatment [26].

The activation of the sympathetic nervous system is involved in CsA-induced hypertension and nephropathy. In the kidneys of CsA-treated rats, the upregulation of tyrosine hydroxylase expression, marker of sympathetic activation, was prevented by empagliflozin administration. These data indicate that the beneficial effects of SGLT2 inhibitors on blood pressure and renal fibrosis in CsA nephropathy are mediated by its action on sympathetic activity in line with results obtained in type 2 diabetes and obesity, characterized by sympathetic overactivity [42].

In addition to diuretic and natriuretic effects, the treatment with SGLT2 inhibitors may modulate the excretion of electrolytes involved in mineral metabolisms [43], as calcium and phosphate. In CsA-treated rats, the significant increase in plasma calcium levels, as compared with control rats, was reverted to control values by empagliflozin administration, which caused a significant increase in urinary calcium excretion as compared to both control and CsA-treated rats.

Urinary phosphate excretion in CsA-treated rats was increased as compared to control values, although plasma phosphate level did not significantly change, and empagliflozin administration in CsA-treated rats blunted the increase in urinary phosphate excretion and reduced plasma phosphate levels. In addition, plasma Tracp-5b levels, used a marker of osteoclast activity, were increased in CsA-treated rats [44, 45] as compared to control rats and showed a slight decrease in CsA + empagliflozin-treated rats. Although the investigation of bone metabolism is outside the aims of our study, these data strongly suggest that SGLT2 inhibitors have a role also in mineral homeostasis [43].

In our experimental conditions, empagliflozin administration results in a beneficial effect on glomerular filtration rate. In fact, CsA treatment caused a slight reduction in glomerular filtration rate, although remaining in normal range, as compared to control rats, which is blunted by empagliflozin administration, indicating an intrarenal protective hemodynamic action caused by SGLT2 inhibition.

The onset of renal fibrosis, promoted by inflammatory cell infiltration, characterized CsA nephropathy in the chronic phase [46, 47]. In our experimental conditions, empagliflozin administration blocked the inflammatory cell infiltration induced by CsA administration, preventing the development of renal fibrosis both at glomerular and at tubulo-interstitial levels. In fact, inflammation plays a pivotal role in the onset and progression of renal fibrosis and the anti-inflammatory effect of empagliflozin could be an important mechanism for nephroprotection, something that could have a protective role also in non-diabetic kidney diseases [26]. Since the experimental design of our protocol consists of the simultaneous administration of CsA and empagliflozin from the beginning of the study, the results obtained suggest that SGLT2 inhibition might have a preventive role against the development of renal diseases that is not restricted, as commonly thought, to diabetic nephropathy.

Taken together, the present data, by demonstrating the beneficial effects of empagliflozin treatments in CsA nephropathy, further suggest that SGLT2 inhibitors might have positive effects also in non-diabetic renal diseases.

## Data Availability

All data generated or analyzed during this study are included in the article and are available from the corresponding Author.

## References

[1] Cohen DJ, Loertscher R, Rubin MF (1984). Cyclosporine: a new immunosuppressive agent for organ transplantation. Ann Intern Med.

[2] Kovarik JM, Burtin P (2003). Immunosuppressants in advanced clinical development for organ transplantation and selected autoimmune diseases. Expert Opin Emerg Drugs Actions.

[3] Chighizola CB, Ong VH, Meroni PL (2017). The use of cyclosporine A in reumathology: a 2016 comprehensive review. Clin Rev Allerg Immunol.

[4] Naesens M, Kuypers DRJ, Sarwal M (2009). Calcineurin inhibitor nephrotoxicity. Clin J Am Soc Nephrol.

[5] Hoskova L, Malek I, Kopkan L (2017). Pathophysiological mechanisms of calcineurin inhibitor-induced nephrotoxicity and arterial hypertension. Physiol Res.

[6] Bennett WM, DeMattos A, Meyer MM (1996). Chronic cyclosporine nephropathy: the Achilles’ heel of immunosuppressive therapy. Kidney Int.

[7] Muller GA, Zeisberg M, Strutz F (2000). The importance of tubulointerstitial damage in progressive renal disease. Nephrol Dial Transpl.

[8] Eddy AA (2014). Overview of the cellular and molecular basis of kidney fibrosis. Kidney Int.

[9] Humphreys BD (2018). Mechanisms of renal fibrosis. Annu Rev Physiol.

[10] Wanner C, Inzucchi SE, Lachin JM (2016). Zinman B for EMPA-REG OUTCOME Investigators. N Engl J Med.

[11] Mosenzon O, Wiviott SD, Cahn A (2019). Effects of dapagliflozin on development and progression of kidney disease in patients with type 2 diabetes: an analysis from the DECLARE-TIMI 58 randomised trial. Lancet Diabetes Endocrinol.

[12] Neal B, Perkovic V, Mahaffey KW (2017). Canagliflozin and Cardiovascular and Renal Events in Type 2 Diabetes. N Engl J Med.

[13] Perkovic V, Jardine MJ, Neal B (2019). Canagliflozin and Renal Outcomes in Type 2 Diabetes and Nephropathy. N Engl J Med.

[14] Neuen BL, Ohkuma T, Neal B (2018). Cardiovascular and renal outcomes with canagliflozin according to baseline kidney function. Circulation.

[15] Heerspink HJL, Kosiborod M, Inzucchi SE (2018). Renoprotective effects of sodium-glucose cotransporter-2 inhibitors. Kidney Int.

[16] Maki T, Maeno S, Maeda Y (2019). Amelioration of diabetic nephropathy by SGLT2 inhibitors independent of its glucose-lowering effect: a possible role of SGLT2 in mesangial cells. Sci Rep.

[17] Nespoux J, Vallon V (2020). Renal effects of SGLT2 inhibitors: an update. Curr Opin Nephrol Hypertens.

[18] Thomas MC, Cherney DZI (2018). The actions of SGLT2 inhibitors on metabolism, renal function and blood pressure. Diabetologia.

[19] Wang XX, Levi J, Luo Y (2017). SGLT2 protein expression is increased in human diabetic nephropathy: SGLT2 protein inhibition decreases renal lipid accumulation, inflammation, and the development of nephropathy in diabetic mice. J Biol Chem.

[20] Wanner C (2017). EMPA-REG outcome: the nephrologist’s point of view. Am J Cardiol.

[21] Bonora BM, Avogaro A, Fadini GP (2020). Extraglycemic effects of SGLT2 inhibitors: a review of the evidence. Diabetes Metab Syndr Obes Targets Ther.

[22] Cassis P, Locatelli M, Cerullo D (2018). SGLT2 inhibitor dapagliflozin limits podocyte damage in proteinuric nondiabetic nephropathy. JCI Insight.

[23] Manne NDPK, Ginjupalli GK, Rice KM (2019). Long-term treatment with empagliflozin attenuates renal damage in obese Zucker rat. Exp Clin Endocrinol Diabetes.

[24] Abbas NAT, Salem AEI, Awad MM (2018). Empagliflozin SGLT2 inhibitor attenuates renal fibrosis in rats exposed to unilateral ureteric obstruction potential role of klotho expression. Naunyn Schmied Arch Pharmacol.

[25] Takeshige Y, Fujisawa Y, Rahman A (2016). A sodium-glucose co-transporter 2 inhibitor empagliflozin prevents abnormality of circadian rhythm of blood pressure in salt-treated obese rats. Hypertens Res.

[26] Castoldi G, Carletti R, Ippolito S (2020). Renal anti-fibrotic effect of sodium glucose cotransporter 2 inhibition in angiotensin II-dependent hypertension. Am J Nephrol.

[27] Ma Q, Steiger S, Anders HJ (2017). Sodium glucose transporter-2 inhibition has no renoprotective effects on non-diabetic chronic kidney disease. Physiol Rep.

[28] Zhang Y, Thai K, Kepecs DM (2016). Sodium-glucose linked cotransporter-2 inhibition does not attenuate disease progression in the rat remnant kidney model of chronic kidney disease. PLoS One.

[29] Castoldi G, di Gioia CRT, Carletti R (2016). Angiotensin type-2 (AT-2)-receptor activation reduces renal fibrosis in cyclosporine nephropathy: evidence for blood pressure independent effect. Biosci Rep.

[30] Elzinga LW, Rosen S, Bennett WM (1993). Dissociation of glomerular filtration rate from tubulointerstitial fibrosis in experimental chronic cyclosporine nephropathy: role of sodium intake. J Am Soc Nephrol.

[31] Li C, Sun BK, Lim SW (2005). Combined effects of losartan and pravastatin on interstitial inflammation and fibrosis in chronic cyclosporine-induced nephropathy. Transplantation.

[32] Castoldi G, di Gioia C, Giollo F (2016). Different regulation of miR-29a-3p in glomeruli and tubules in an experimental model of angiotensin II-dependent hypertension: potential role in renal fibrosis. Clin Exp Pharmacol Physiol.

[33] Mulder J, Hokfelt T, Knuepfer MM (2013). Renal sensory and sympathetic nerves reinnervate the kidney in a similar time-dependent fashion after denervation in rats. Am J Physiol Regul Integr Comp Physiol.

[34] Murray BM, Paller MS, Ferris TF (1985). Effect of cyclosporine administration on renal hemodynamics in conscious rats. Kidney Int.

[35] Ciresi DL, Lloyd MA, Sandberg SM (1992). The sodium retaining effects of cyclosporine. Kidney Int.

[36] Shimizu H, Kumai T, Kobayashy S (2001). Involvement of tyrosine hydroxylase upregulation in cyclosporine-induced hypertension. Jpn J Pharmacol.

[37] Moss NG, Powell SL, Falk RJ (1985). Intravenous cyclosporine activates afferent and efferent renal nerves and causes sodium retention in innervated kidneys in rats. Proc Natl Acad Sci USA.

[38] DeFronzo RA, Norton L, Abdul-Ghani M (2017). Renal, metabolic and cardiovascular considerations of SGLT2 inhibition. Nat Rev Nephrol.

[39] Baker WL, Buckley LF, Kelly MS (2017). Effects of sodium-glucose cotransporter 2 inhibitors on 24-hour ambulatory blood pressure: a systematic review and meta-analysis. J Am Heart Assoc.

[40] Mancia G, Cannon CP, Tikkanen I (2016). Impact of empagliflozin on blood pressure in patients with type 2 diabetes mellitus and hypertension by background antihypertensive medication. Hypertension.

[41] Mazidi M, Rezaie P, Gao HK (2017). Effect of sodium-glucose cotransport-2 inhibitors on blood pressure in people with type 2 diabetes mellitus: a systematic review and meta-analysis of 43 randomized control trials with 22 528 patients. J Am Heart Assoc.

[42] Matthews VB, Elliot RH, Rudnicka C (2017). Role of the sympathetic nervous system in regulation of the sodium glucose cotransporter 2. J Hypertens.

[43] Cianciolo G, De Pascalis A, Capelli I (2019). Mineral and electrolyte disorders with SGLT2i therapy. JBMR Plus.

[44] Spolidorio LC, Herrera BS, Coimbra LS (2010). Intermittent therapy with 125 vitamin D and calcitonin prevents cyclosporin-induced alveolar bone loss in rats. Calcif Tissue Int.

[45] Chen RY, Fu MM, Chih YK (2011). Effect of cyclosporine-A on orthodontic tooth movement in rats. Orthod Craniofac Res.

[46] Araujo LP, Truzzi RR, Mendes GEF (2012). Annexin A1 protein attenuates cyclosporine-induced renal hemodynamics changes and macrophage infiltration in rats. Inflamm Res.

[47] Jin M, Lv P, Chen G (2017). Klotho ameliorates cyclosporine A-induced nephropathy via PDLIM2/NF-kB p65 signaling pathway. Biochem Biophys Res Commun.

